# Supplementary material for: Intrinsic cellular persistent firing sustains hippocampal spatial representations during working memory
Source: Sci Adv. 2026 Oct 30;12(44):eaef9907. doi: 10.1126/sciadv.aef9907 (PMC13721955; doi:10.1126/sciadv.aef9907)
Supplement: Supplementary file 1 [file sciadv.aef9907_sm.pdf]

Science Advances
AAAS

Supplementary Materials for

Intrinsic cellular persistent firing sustains hippocampal spatial representations during working memory

Babak Saber Marouf *et al.*

Corresponding author: Motoharu Yoshida, motoharu.yoshida@lin-magdeburg.de

Sci. Adv. **12**, eaef9907 (2026)
DOI: 10.1126/sciadv.aef9907

This PDF file includes:

Figs. S1 to S8

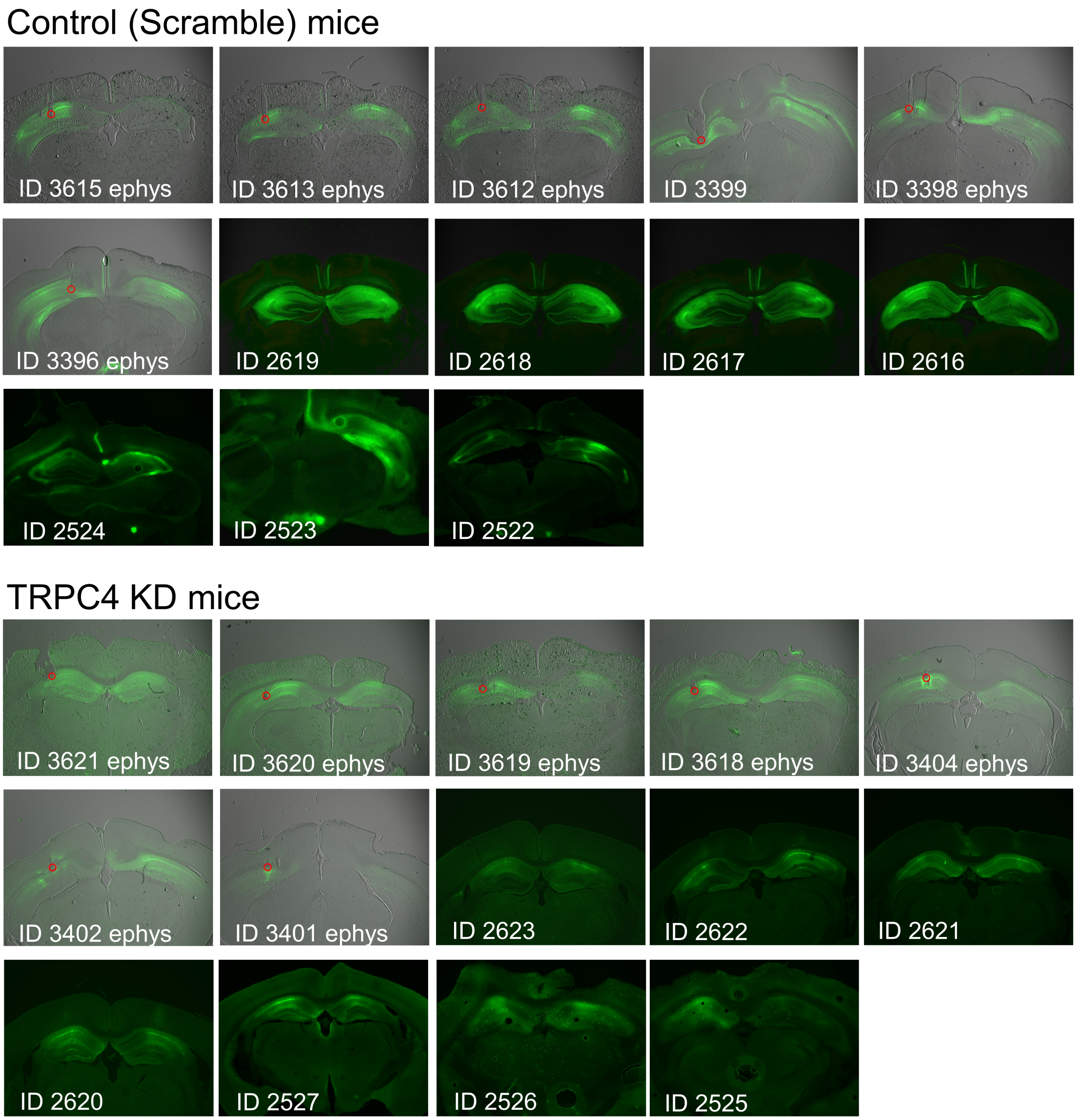

Figure S1. Virus expression and tetrode track. GFP expression and track locations for each animal in both the control and TRPC4 KD groups. Animal IDs followed by “ephys” indicate that those mice were used for electrophysiological recording. The tips of the tracks are highlighted with a red circle. Other animals were only used for the behavioral test. The image for animal ID 2621 is shown as an example in Fig. 1.

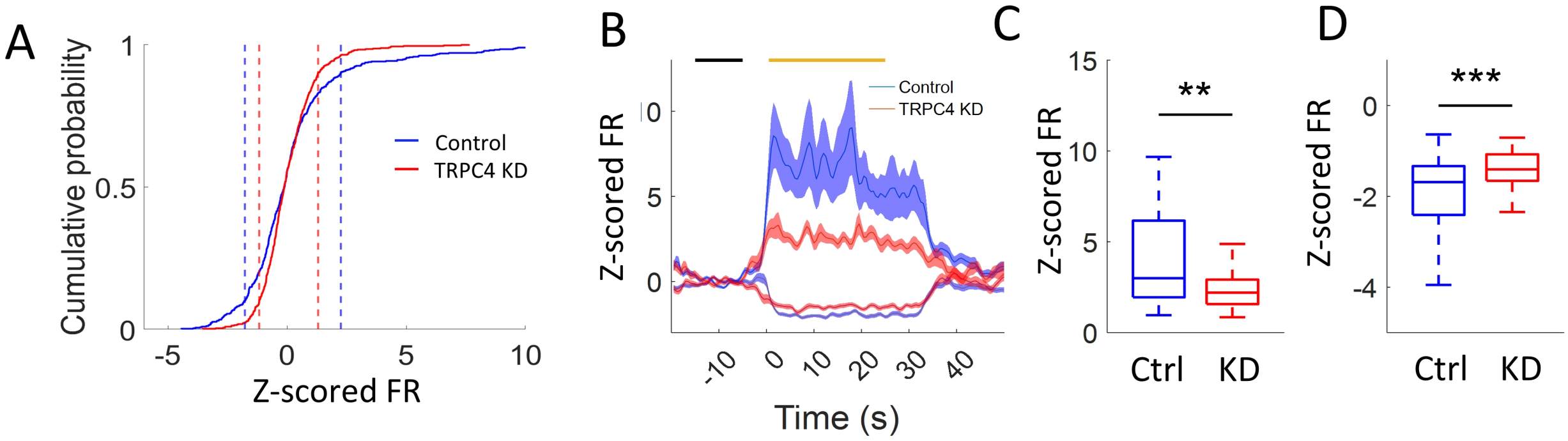

Figure S2. Distribution of the mean activity during the delay period and the activity of delay-activated (Act) and delay-suppressed (Sup) cells. (**A**) Cumulative distribution function of the mean activity during the delay period for all recorded cells. The dotted vertical lines indicate the 10th and 90th percentile crossings for the control (blue) and TRPC4 KD (red) groups. The interdecile (10th–90th percentile) ranges were 4.03 and 2.48 for the control and KD groups, respectively. The interquartile ranges were 1.65 and 1.25 for the control and KD groups, respectively. The variances were 41.02 and 1.43 for the control and KD groups, respectively (Brown–Forsythe test, $F(1, 1080) = 12.82$, $p < 0.001$, n = 533 and 549), and were 1.61 and 0.78 for the control and KD groups, respectively, after removing outliers based on Tukey's outlier criterion (Brown–Forsythe test, $F(1, 1014) = 42.25$, $p < 0.001$; see Methods). (**B**) Average z-scored firing rate (FR) of the delay-activated (Act) and delay-suppressed (Sup) cells shown in Fig. 2E. The black and orange horizontal lines at the top indicate the baseline period and the delay period, respectively. (**C**) Comparison of the z-scored firing rates of the Act cells in B averaged over the delay period (0–25 s; WRS test, $p = 0.0077$, n = 80 and 43 for the control and KD groups, respectively). (**D**) Comparison of the z-scored firing rates of the Sup cells in B averaged over the delay period (0–25 s; WRS test, $p < 0.001$, n = 118 and 64 for the control and KD groups, respectively).

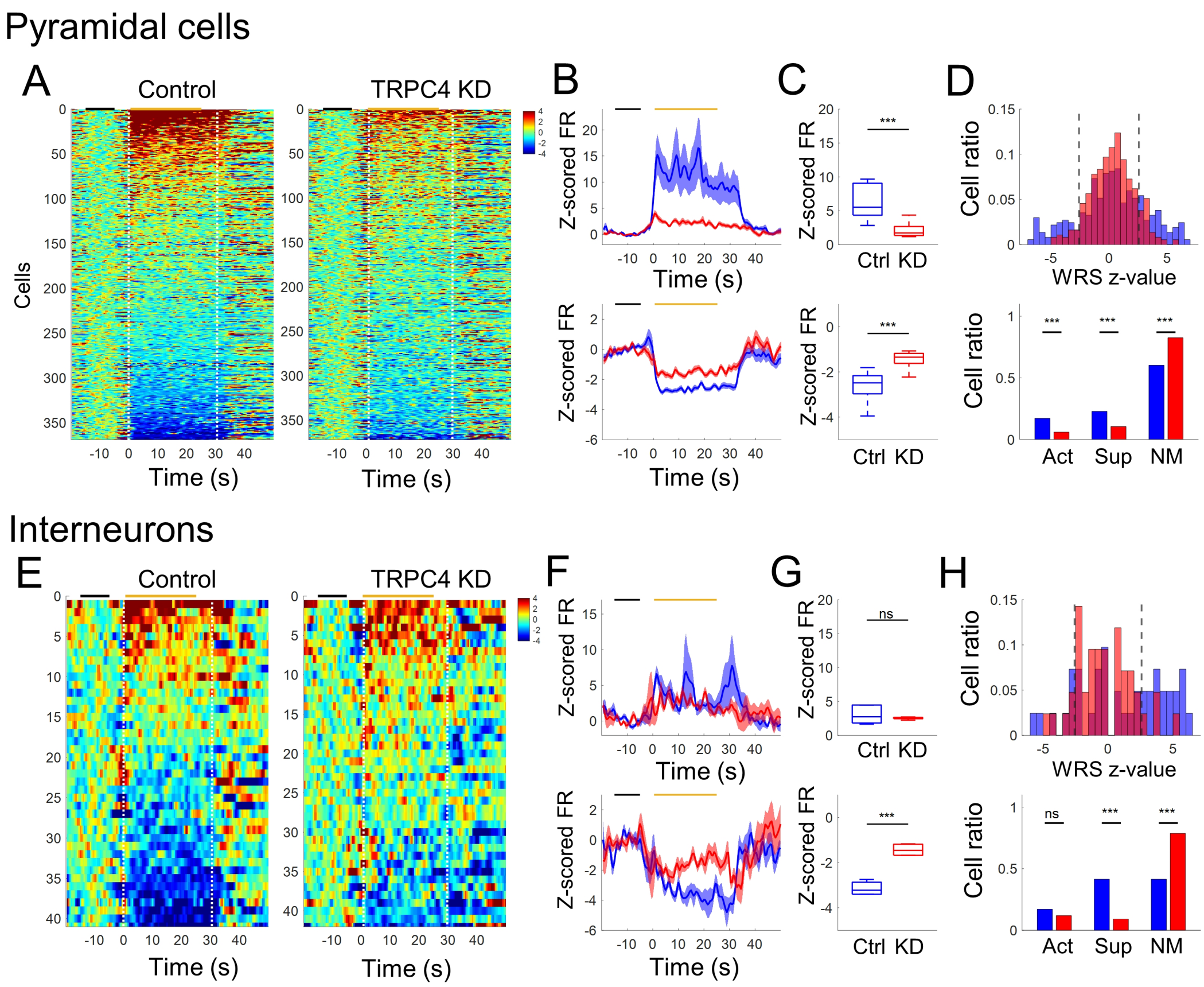

Figure S3. Activity of putative pyramidal and interneurons. (**A**) Z-scored firing rate of putative pyramidal cells sorted by their delay period activity. Black and orange horizontal lines at the top of each panel indicate the baseline period and the period for cell sorting, respectively. (**B**) Average z-scored firing rate (FR) of the top (upper panel) and bottom (lower panel) 10% cells in A. (**C**) Comparison of z-scored firing rate of the top (upper panel; Wilcoxon rank-sum test, $p < 0.001$) and bottom (lower panel; Wilcoxon rank-sum test, $p < 0.001$) 10 % cells in A during the delay period (0-25 s). (**D**) Classification of activated (Act), suppressed (Sup) and non-modulated (NM) cells using the Mann–Whitney U (MWU) test. The upper panel shows the distribution of Wilcoxon rank-sum z-values and the lower panel shows the ratio of cells in each category (WRS test, $p < 0.001$ for all groups). (**E to H**) Same as A to D but for putative interneurons. Statistical tests in G: WRS test, $p = 0.6905$ in the upper panel, and $p < 0.001$ in the lower panel.

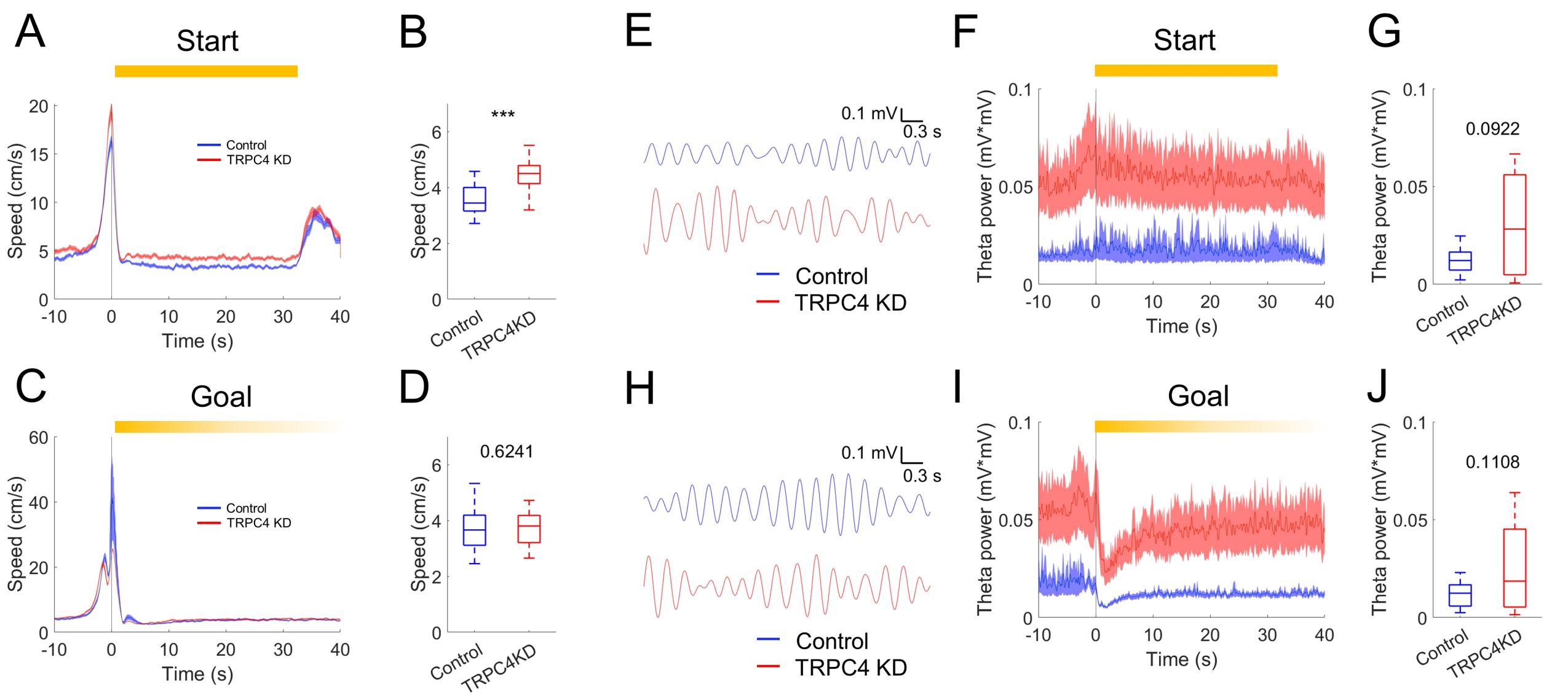

Figure S4. Running speed and theta power at the start and goal areas. (**A**) Average running speed of control and TRPC4 KD animals at the start area. Time is relative to entry into the start area. This figure includes both the sample (ITI) and choice (delay) phases. (**B**) Comparison of the speed in the start area calculated for each session (Wilcoxon rank-sum test, $p < 0.001$; n = 25 and 28 sessions). (**C**) Same as A but for the goal area. Note that there was no set duration during which animals stayed at the goal area. While we show 40 s of goal speed data, animals were already out of the goal areas in some trials. (**D**) Comparison of the speed in the goal area across all sessions (Wilcoxon rank-sum test, $p = 0.62$; n = 25 and 28 sessions). For this comparison, the speed of each session was calculated using the entire time the animal spent in the goal areas in each session. (**E**) Examples of filtered theta oscillations at the start area. (**F**) Theta power of the control and TRPC4 KD animals at the start area. (**G**) Comparison of the averaged theta power in the start area calculated in each session (Wilcoxon rank-sum test, $p = 0.092$; n = 25 and 28 sessions). (**H**) Examples of filtered theta oscillations at the goal area. (**I**) Theta power of control and TRPC4 KD animals at the goal area. As in C, animals were out of the goal area in some trials before the end of the trace. (**J**) Comparison of the theta power in the goal area (Wilcoxon rank-sum test $p = 0.11$; n = 25 and 28 sessions). As in D, the theta power of each session was calculated using the entire time the animal spent in the goal area.

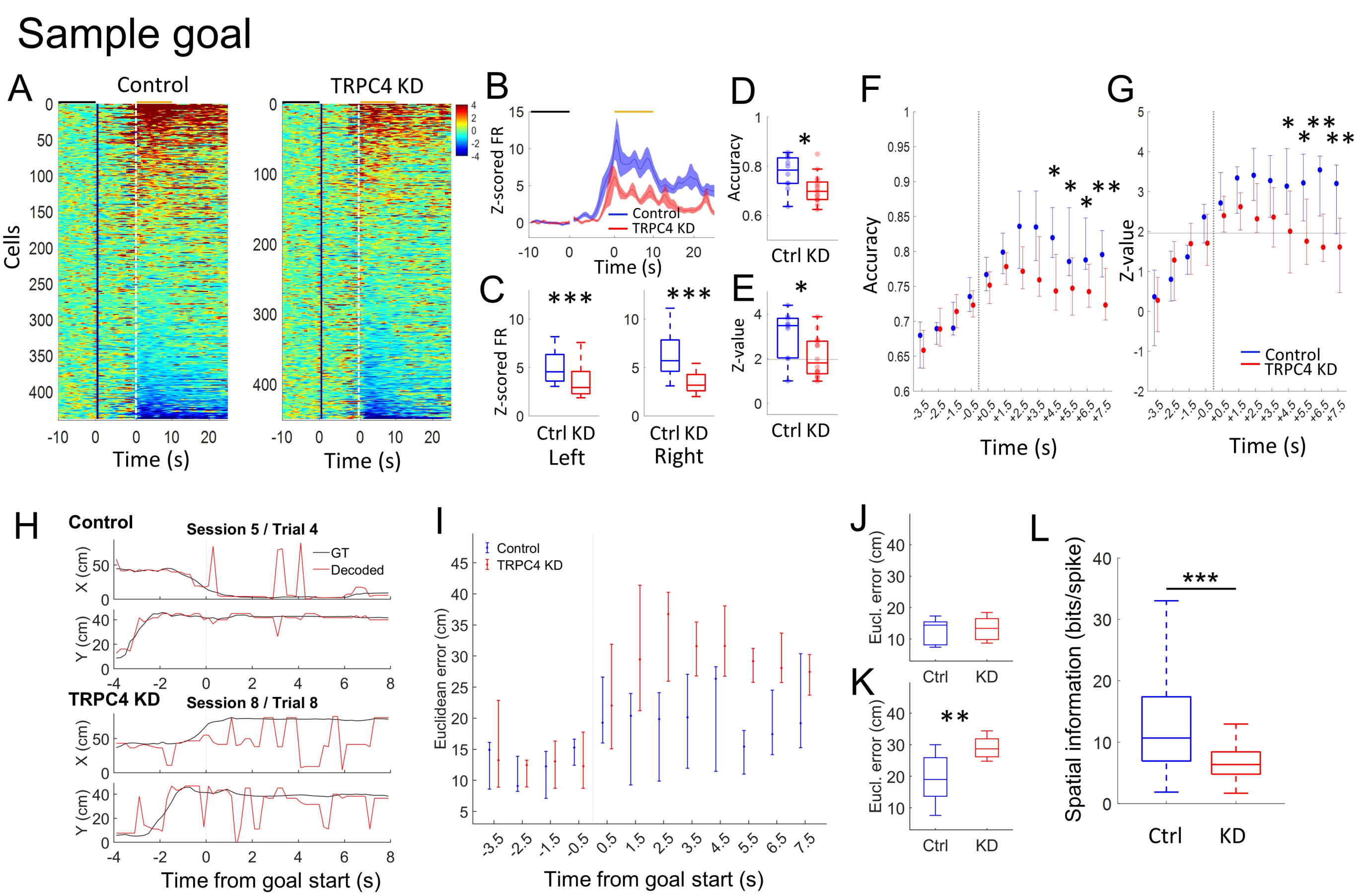

Figure S5. Persistent activity and spatial representation at sample goals. (**A**) Z-scored firing rate of cells sorted by their activity during the 10 s goal period similar to Fig. 7A but only using the sample phase. (**B**) Activity of the top 10% cells in A. (**C**) Comparison of the activity of the top 10% cells during the 10 s goal period (0-10 s) at the left and right goals (WRS test, $p < 0.001$ and $p < 0.001$, respectively). (**D-E**) The accuracy (**D**) and z-value (**E**) of the SVM prediction of left vs. right goals based on the activity of all cells in individual sessions during the 10 s sample goal period (WRS test, $p = 0.021$ and 0.019 for D and E, respectively). (**F-G**) The accuracy (**F**) and z-value (**G**) of the SVM prediction of left vs. right goals at different time periods before and during the 10 s goal period. (**H**) Example real (GT) and decoded X and Y positions of mice from 4 s before to 8 s after goal entry. (**I**) Decoding error measured as distance between true and estimated positions at different time periods before and during the goal period. (**J and K**) Comparison of error distance between the control and the KD groups before (-4 to 0 s) and after (0 to 8 s) goal entry (WRS test, $p = 0.60$ and 0.0046, respectively). (**L**) Spatial information of goal cells as in Fig. 6K but only in sample phase (WRS test, $p < 0.001$).

Choice goal

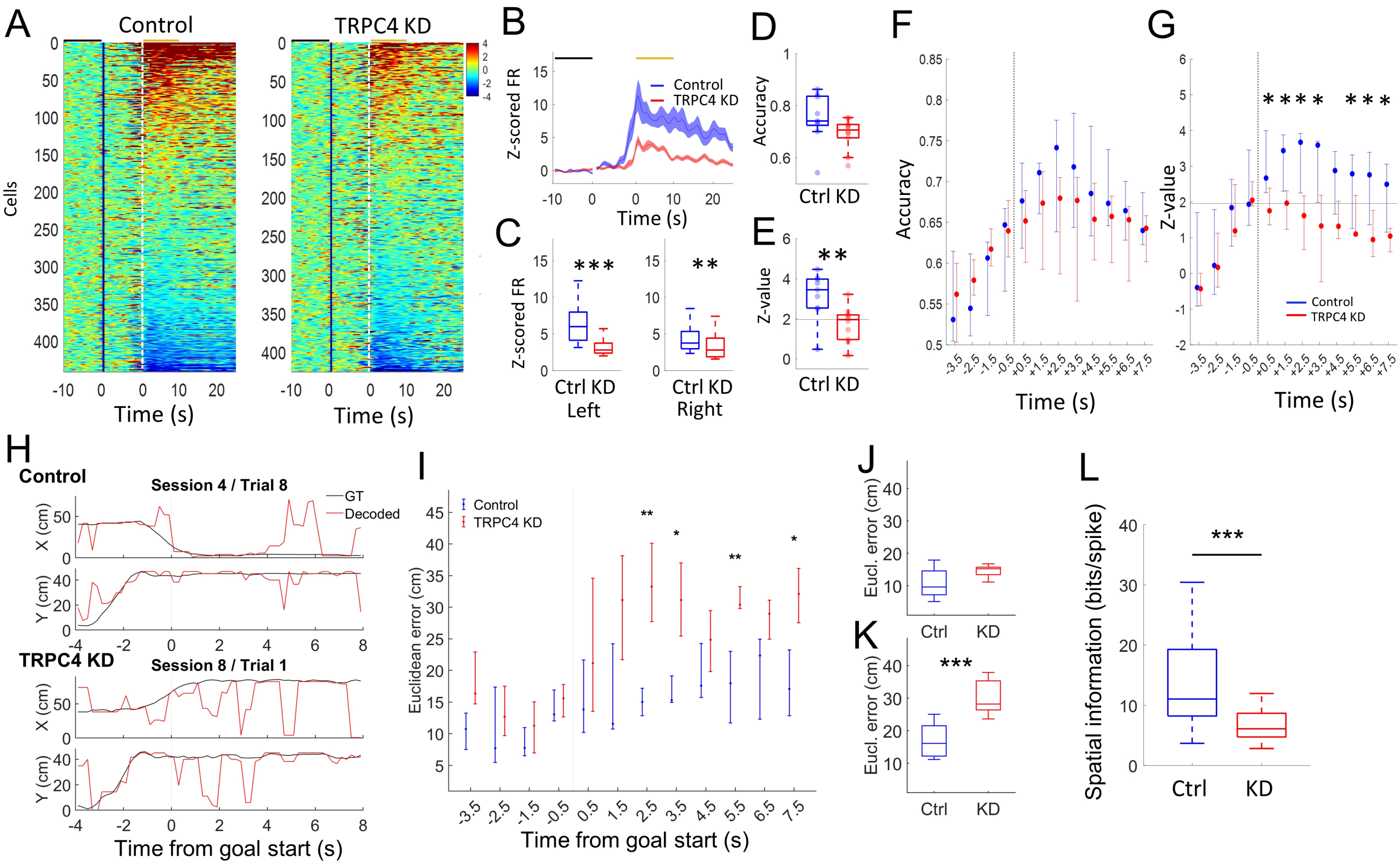

Figure S6. Persistent activity and spatial representation at choice goals. (**A**) Z-scored firing rate of cells sorted by their activity during the 10 s goal period similar to Fig. 7A but only using choice phase. (**B**) Activity of the top 10% cells in A. (**C**) Comparison of the activity of the top 10% cells during the 10 s goal period (0-10 s) at the left and right goals (WRS test, $p < 0.001$ and $p = 0.0016$, respectively). (**D-E**) The accuracy (**D**) and z-value (**E**) of the SVM prediction of left vs. right goals based on the activity of all cells in individual sessions during the 10 s choice goal period (WRS test, $p = 0.057$ and 0.054 in D and E, respectively). (**F-G**) The accuracy (**F**) and z-value (**G**) of the SVM prediction of left vs. right goals at different time periods before and during the 10 s goal period. (**H**) Example real (GT) and decoded X and Y positions of mice from 4 s before to 8 s after goal entry. (**I**) Decoding error measured as distance between true and estimated positions at different time periods before and during the goal period. (**J and K**) Comparison of error distance between the control and the KD groups before (-4 to 0 s) and after (0 to 8 s) goal entry (WRS test, $p = 0.11$ and $p < 0.001$, respectively). (**L**) Spatial information of goal cells as in Fig. 6K but only in choice phase (WRS test, $p < 0.001$).

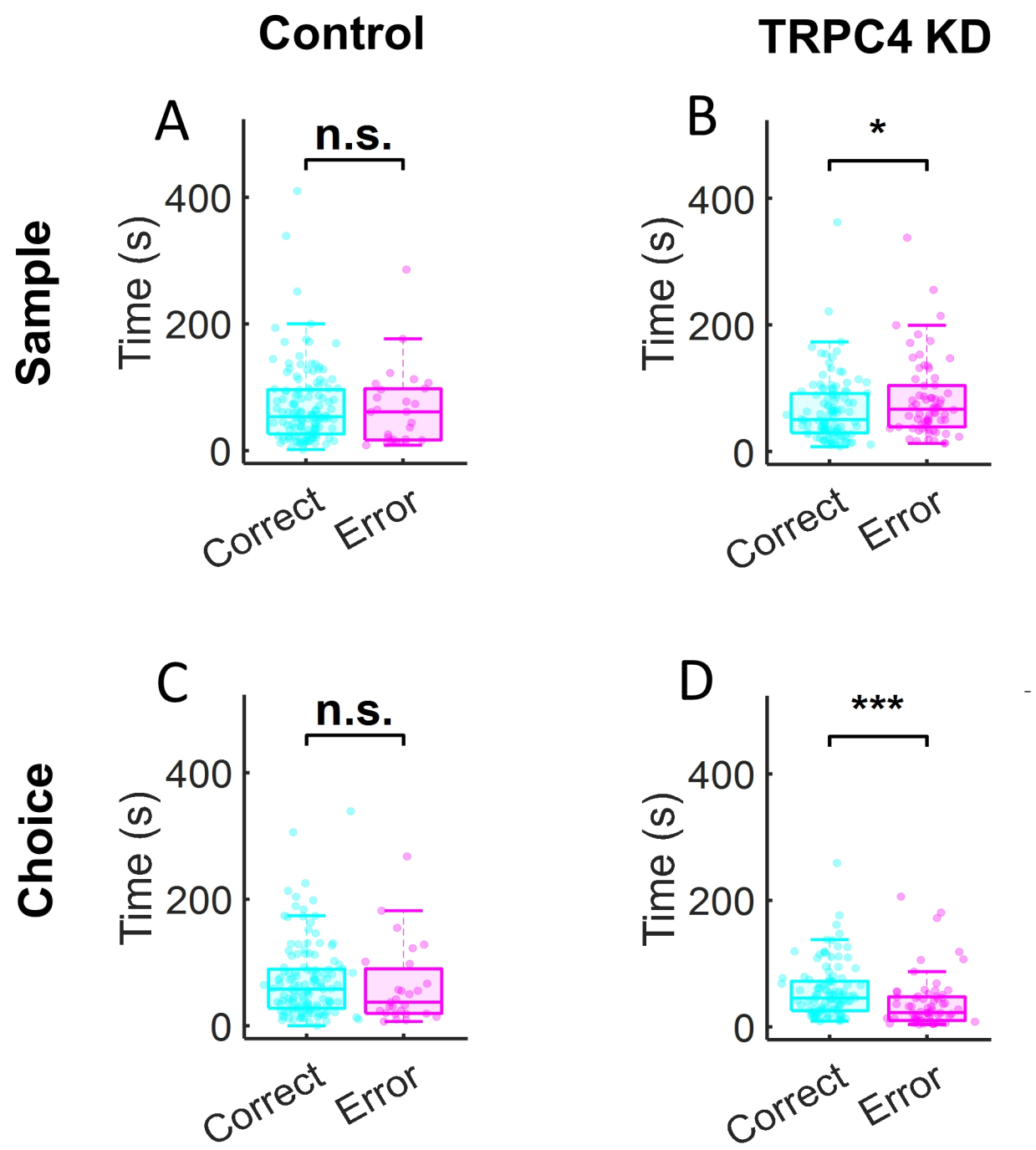

Figure S7. Comparison of time spent at the goals in correct and error trials. The time animal spent at the goal in sample and choice phases was measured separately in each trial. Each dot corresponds to a trial. (**A**) Sample goal duration in control mice (WRS test, $p = 0.49$; $n = 132$ correct, $n = 27$ error trials). (**B**) Sample goal duration in TRPC4 KD mice (WRS test, $p = 0.049$; $n = 111$ correct, $n = 68$ error trials). (**C**) Choice goal duration in control mice (WRS test, $p = 0.20$; $n = 132$ correct, $n = 27$ error trials). (**D**) Choice goal duration in TRPC4 KD mice (WRS test, $p < 0.001$; $n = 111$ correct, $n = 68$ error trials).

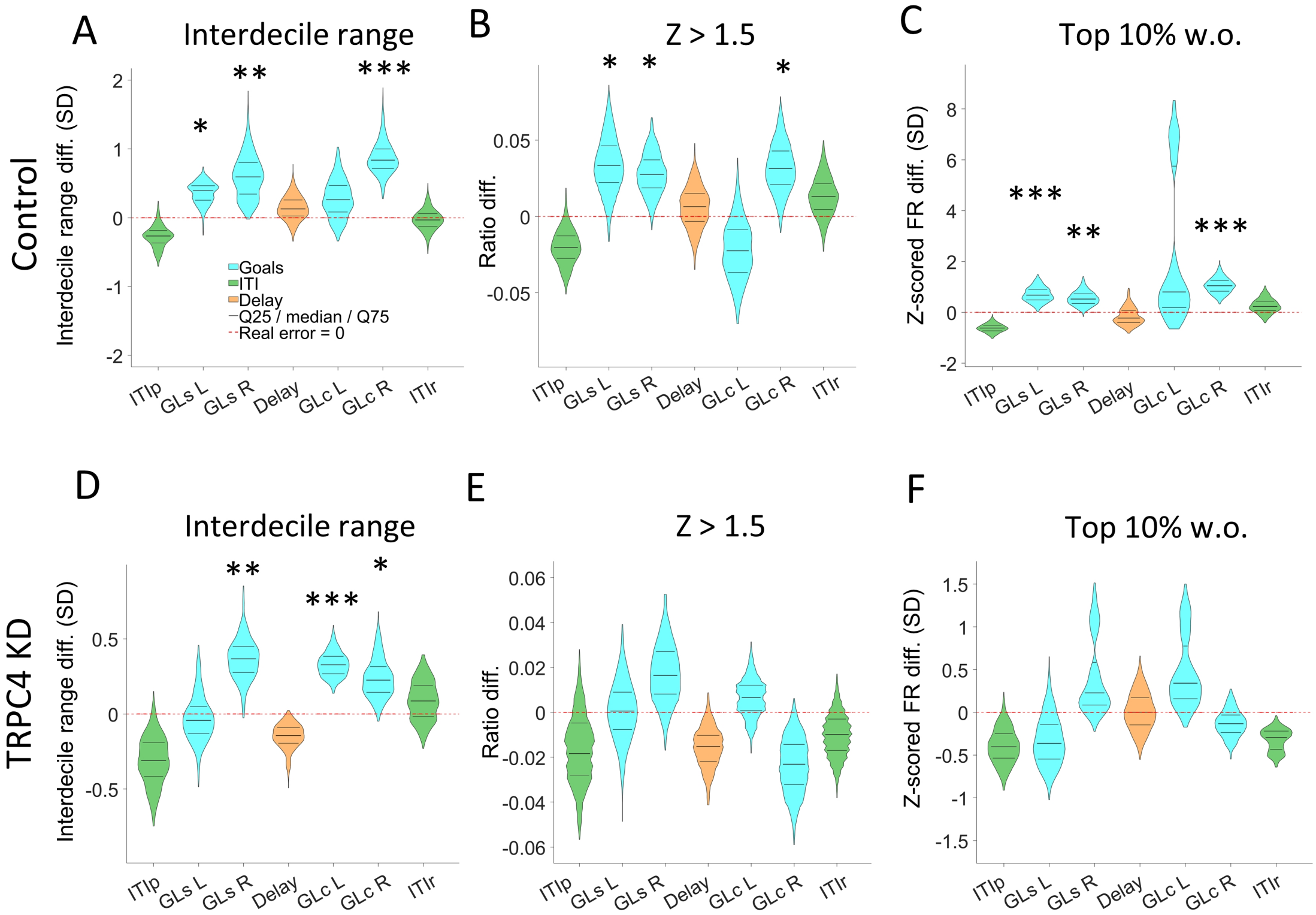

Figure S8. Differences in cellular activity between correct and error trials across task phases. In each panel, the bootstrap distributions of the metric values from correct trials are shown relative to the metric value calculated from the error trials (indicated by the horizontal red line at y = 0; see Methods). Therefore, the y-axis represents the difference (diff.) in metric values between correct and error trials, and violin plots above the red line indicate that the metric value obtained from error trials was smaller than that of correct trials. Comparison were made at five different phases: ITI before the trial (ITIp), left (L) and right (R) sample goals (GLs), delay, left (L) and right (R) choice goals (GLc), and ITI after the trial (ITIr). (**A-C**) Data from control mice. (**A**) Interdecile (10th–90th percentile) range (p = 0.96 , 0.016, 0.0030, 0.20, 0.16, < 0.001, 0.60 from left to right). (**B**) Ratio of cells with a z-scored firing rate larger than 1.5 SD (p = 0.96, 0.028, 0.013, 0.33, 0.89, 0.019, 0.14 from left to right). (**C**) Average z-scored firing rate of the top 10% most active cells without outlier cells (w.o.; p = 1.0, < 0.001, 0.0070, 0.69, 0.18, < 0.001, 0.18 from left to right). (**D-F**) Data from TRPC4 KD mice. (**D**) Interdecile (10th–90th percentile) range (p = 0.97, 0.63, 0.0040, 0.99, < 0.001, 0.011, 0.29 from left to right). (**E**) Ratio of cells with a z-scored firing rate larger than 1.5 SD (p = 0.86 , 0.49, 0.083, 0.97, 0.18, 0.98, 0.85 from left to right). (**F**) Average z-scored firing rate of the top 10% most active cells without outlier cells (p = 0.97, 0.86, 0.11, 0.50, 0.073, 0.80, 1.0 from left to right).
